# Application of nano-carbon and titanium clip combined labeling in robot-assisted laparoscopic transverse colon cancer surgery

**DOI:** 10.1186/s12893-021-01248-6

**Published:** 2021-05-24

**Authors:** Nan Lin, Jiandong Qiu, Junchuan Song, Changwei Yu, Yongchao Fang, Weihang Wu, Weijin Yang, Yu Wang

**Affiliations:** 1 Department of General Surgery, 900 Hospital of the Joint Logistics Team, Fuzhou, China; 2 Department of Oncological Surgery, Sanming First Hospital Affiliated to Fujian Medical University, Fuzhou, China; 3 Department of General Surgery, Dongfang Hospital, Xiamen University, Xiamen, China; 4 Clinical Institute of Fuzhou General Hospital, Fujian Medical University, Fuzhou, China

**Keywords:** Transverse colon cancer, Robot-assisted surgery, Colonoscopy, Nano-carbon

## Abstract

**Background:**

Robot-assisted laparoscopic transverse colon tumor surgery requires precise tumor localization. The purpose of this study was to evaluate the safety and efficacy of nano-carbon and titanium clip combination labeling methods in robot-assisted transverse colon tumor surgery.

**Methods:**

From January 2018 to January 2019, the clinical data of 16 patients who come from FuZhou, China underwent preoperative nano-carbon and titanium clip combined with robot-assisted laparoscopic transverse colon cancer surgery were retrospectively analyzed.

**Results:**

Of the 16 patients, no signs of abdominal pain, fever, or diarrhea were observed after colonoscopy. Two titanium clips were seen on all of the 16 patients' abdominal plain films. Nano-carbon staining sites were observed during the operation, and no staining disappeared or abdominal cavity contamination. All patients underwent R_0_ resection. The average number of lymph nodes harvsted was 18.23 ± 5.04 (range, 9–32). The average time to locate the lesion under the laparoscopic was 3.03 ± 1.26 min (range, 1–6 min), and the average operation time was 321.43 ± 49.23 min (range, 240–400 min). All were consistent with the surgical plan, and there was no intraoperative change of surgical procedure or conversion to open surgery.

**Conclusion:**

Preoperative colonoscopy combined with nano-carbon and titanium clip is safe and effective in robot-assisted transverse colon cancer surgery. A At the same time, the labeling method shows potential in shortening the operation time, ensuring sufficient safety margin and reducing complications.

## Background

Colorectal cancer is the third most commonly diagnosed cancer in males and is the second most commonly diagnosed cancer in females [1]. In recent years, with the improvement of people's living standards and dietary habits, the incidence of colon cancer is rising [2]. Radical resection is the only cure treament for local colon cancer. In the past few decades, with the application of laparoscopy, the colorectal surgery has undergone significant changes. Compared with traditional open surgery, laparoscopic techniques allow improved visualization of areas difficult to reach by means of open surgery, and thus more precise dissection of anatomic structures [3], besides, laparoscopic surgery offers many advantages, such as minimizing surgical trauma, reducing blood loss, reducing postoperative pain, and promoting recovery [4–6]. Although laparoscopic surgery has many advantages, it still has some visual and operational limitations. Da Vinci robotic assisted surgery is an emerging minimally invasive technique that increases flexibility, improves surgical field of vision, and achieves optimal ergonomics [3]. However, for robotic surgery, accurate tumor localization is the key to the success of robotic colon surgery due to the lack of effective tactile feedback assistance, especially when the tumor locates in the transverse colon.

Currently, many methods are used for preoperative localization of colon tumors, including double-contrast barium enema, computed tomography colonography, titanium clip positioning, intraoperative colonoscopy, and preoperative injection stain positioning. Common staining agents for dyeing include methylene blue, indigo carmine, phthalocyanine green and Indian ink [7–10]. However, the above methods have their own shortcomings and limitations, such as radioactivity, inaccurate positioning, easy removal of titanium clips, and dispersion of stains [10–12]. In recent years, with the development of nanotechnology, carbon nanoparticles have been applied to tumor markers, such as colorectal cancer [13], breast cancer [14]. The injected carbon nanoparticle suspension contains nanometer carbon particles with an average diameter of 150 nm. Due to molecular size and permeability, this ensures that these particles do not enter the blood circulation and have no toxic side effects on human body. Since 2007, China Food and Drug Administration approved the use of nano-carbon suspension in human.

In this study, we use nano-carbon and titanium clip combined labeling method to locate transverse colon tumor. Then, we performed robotic-assisted transverse colon tumor surgery and aimed to evaluate the safety and effectiveness of the approach in robotic-assisted surgery.

## Methods

### Patients

We retrospectively assessed 16 patients who were candidates for robot-assisted radical resection of transverse colon cancer at the General Surgery of the 900 Hospital of the Joint Logistics Support Force (FuZhou,China) from January 2018 to January 2019. Among them, 5 patients were female and 11 patients were male. All patiens were met the following inclusion criteria, including age of 18–70 years old,had positive colonoscopic results for single transverse colon cancer,were stage I -III according to the TNM, no distant metastasis and no history of abdominal operations. Exclusion criteria included the following: patients before preoperative neoadjuvant chemoradiotherapy, benign tumors, had distant metastasis and patients who underwent emergency surgery, due to obstruction or perforation of the bowel. The surgeries were performed by the same team of surgeons. All surgical methods performed in this study were in accordance with the colorectal cancer guidelines of the National Comprehensive Cancer Network (NCCN). The tumor-node-metastasis (TNM) staging was based on the seventh edition of the American Joint Committee on Cancer staging classification.

This study was approved by the Ethics Committee of the 900 Hospital of the Joint Logistics Support Force. Need for written informed consent was waived owing to the retrospective nature of the study.

### Materials

Carbon nacoparticles suspension injection (Canaline) with a diameter of 150 nm was purchased from Chongqing LUMMY Pharmaceutical Co, Ltd.(Chongqing, China). Disposable injection needle (NM-200U-0423, Olympus, Japan), Rotary Titanium Clip Pusher ( HX-5QR-1, Olympus, Japan) and Metal Titanium Clips (EZ Clip, HX-610-135 L, Olympus, Japan).

### Surgical procedure

**Fig. 1:**
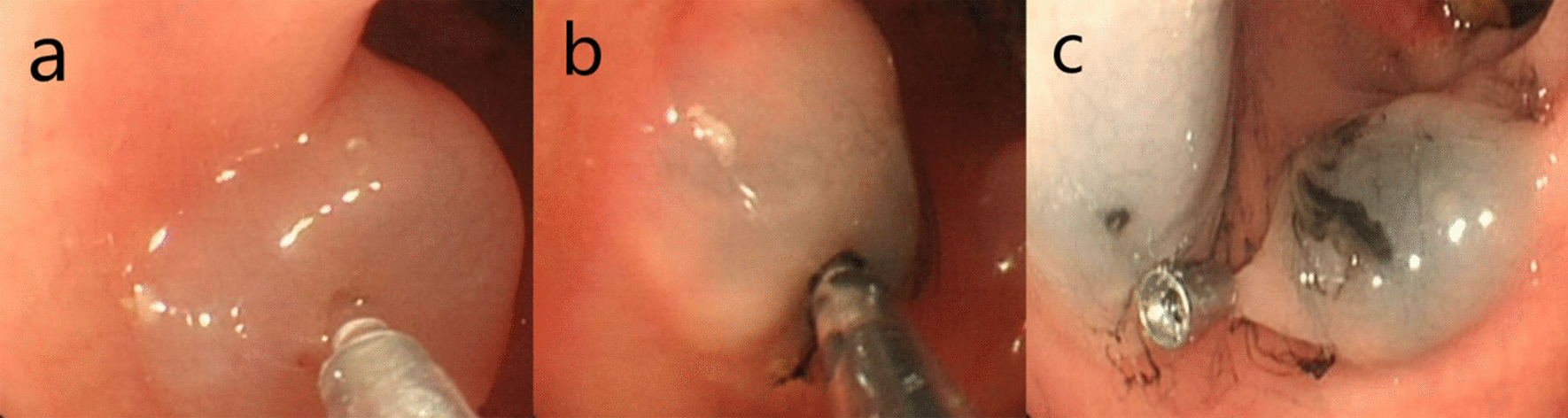
Nano-carbon and titanium clip combination labeling methods under colonoscopy. **a** Injection 1 ml of physiological saline into the submucosa layer to form a suitable submucosal apophysis. **b** Injection of 0.1 ml nanocarbon into the submucosal apophysis. **c** Titanium clip placement

All patients underwent a standard mechanical bowel preparation the day before surgery. A team of 2 experienced endoscopists performed all colonoscopy procedures in the endoscopy centres affiliated with the 900 Hospital of the Joint Logistics Support Force. After a disposable injection needle (NM-200U-0423, Olympus, Japan) was used to inject 1 ml saline into the submucosa under colonoscopy, a suitable submucosal apophysis was created. 0.1 ml of nano-carbon was injected into the submucosal apophysis using another 1-ml syringe. The nano-carbon syringe was removed and replaced with a first syringe containing saline, and the pinhole was washed with 1 mL of saline to flush the nano-carbon remaining (Fig. 1).

**Fig. 2:**
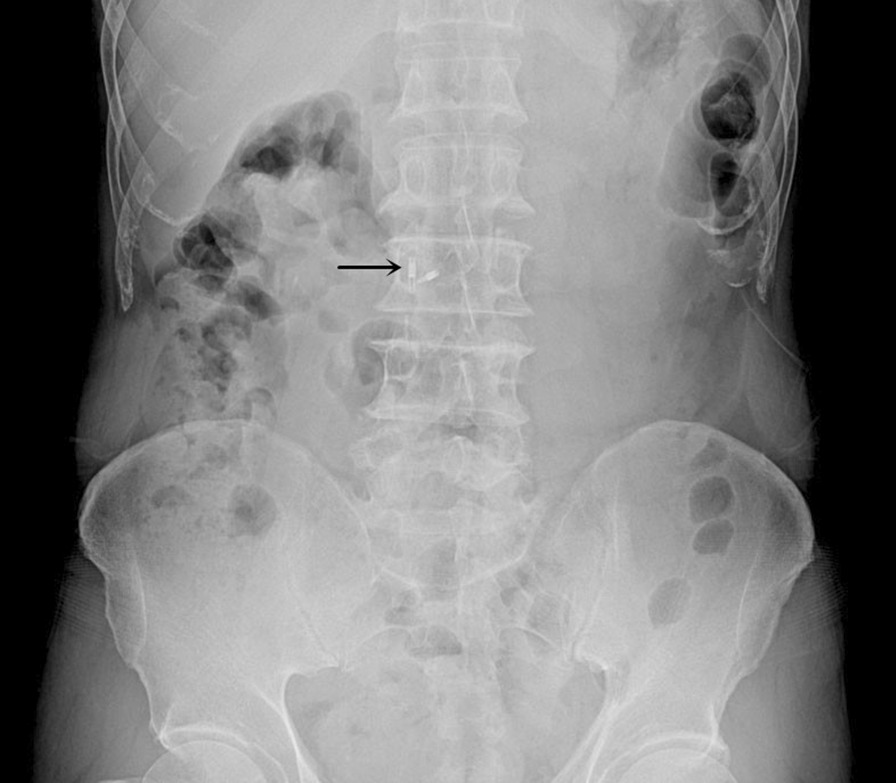
Two titanium clips (black arrow) visible in the flat piece of the abdomen

The nano-carbon is injected at an angle of 90° and at a distance of 1 cm from the edge of the tumor [15]. Two titanium clips (EZ Clip, HX-610-135 L, Olympus, Japan) were individually placed anal-side and oral-side of the tumor from the edge of the tumor. Immediately after titanium clips placed, a plain abdominal X-ray was taken to confirm the position of the titanium clip (Fig. 2). All patients underwent pre-operative positioning 1–7 days before surgery.

## Results

A total of 16 patients were included in the study, including 11men (68.8%) and 5 women (31.2%), with an average age of 59.13 ± 7.42 years. Patient characteristics and tumor characteristics are shown in Table 1. No patients were found to have abdominal discomfort, fever, diarrhea, etc. after receiving colonoscopy. Two titanium clips were seen on all of the 16 patients' abdominal plain films.

**Table 1:** Patient characteristics and tumor characteristics

Variables	No. of patients (%)
Gender	
Male	11 (68.75%)
Female	5 (31.25%)
Mean age (years)	59.13 ± 7.42
Tumor staging(TNM)	
T stage	
Tis	1 (6.25%)
T1	3 (18.75%)
T2	6 (37.50%)
T3	4 (25.00%)
T4	2 (12.50%)
N stage	
N0	9 (56.25%)
N1	6 (37.50%)
N2	1 (6.25%)

**Fig. 3:**
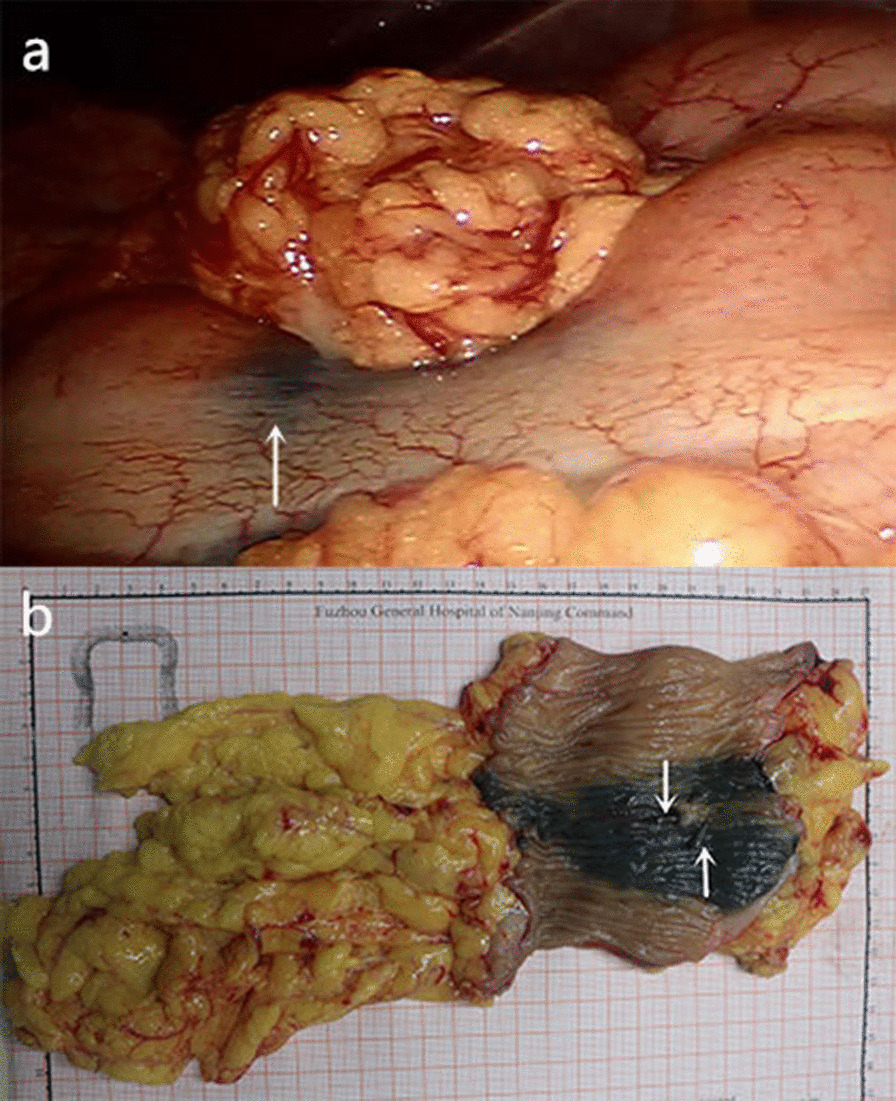
**a** Serosal appearance of colonic lesion labeled by nano-carbon in robot-assisted surgery (The white arrow points to the nano-carbon black stained area). **b** Mucosal appearance of the surgical removal specimen, which can observe nano-carbon black stained area and two titanium clips (white arrow).

All patients underwent robot-assisted surgery, including 5 (31.25%) transverse colon resection, 3 (18.75%) right colectomy, and 8 (50.00%) left colectomy, as shown in Table 2. Nano-carbon staining sites were observed during the operation, and no staining disappeared or abdominal cavity contamination (Fig. 3). All patients in this study underwent R_0_ resection. The number of lymph nodes harvsted in 16 patients was 18.23 ± 5.04 (range, 9–32). The average time to locate the lesion under the laparoscopic was 3.03 ± 1.26 min (range, 1–6 min), and the average operation time was 321.43 ± 49.23 min (range, 240–400 min). All were consistent with the preoperative surgical plan, there was no intraoperative change of surgical procedure or conversion to open surgery. One patient developed postoperative intestinal obstruction and was discharged successfully after conservative treatment for 12 days.One patient developed pulmonary infection due to her advanced age and late post-operative activities.

**Table 2:** Clinical results of nano-carbon and titanium clip markers

Variables	No. of patients (%)	
Surgical approach		
Transverse colectomy	5 (31.25%)	
Right hemicolectomy	3 (18.75%)	
Left hemicolectomy	8 (50.00%)	
Tumor localization by nano-carbon		
Precise	16	
Dyeing dispersion	0	
Dyeing disappears	0	
Postoperative complications		
Bleeding	0	
Anastomotic fistula	0	
Intestinal obstruction	1 (6.75%)	
Infection	1 (6.75%)

Postoperative pathology confirmed that the surgical margins were negative, and no residual carbon particles were observed. The average length of the proximal margin was 6.40 ± 3.29 cm (range, 3.9–18.0 cm), and the distal margin was 9.97 ± 3.89 cm (range 5.5–21.0 cm).

## Discussion

In 2002, Hashizume and Weber et al. first reported robotic colectomy [16, 16]. Since then, more and more studies have shown that robotic colorectal surgery has similar oncological results compared with laparoscopic or open surgery [18, 18], the application of robotic surgery in the field of general surgery has increased year by year. Although laparoscopy has now become the gold standard for a variety of relatively easy general surgery. However, laparoscopic ergonomics and technical limitations, the loss of anatomical orientation due to two-dimensional views make the identification of important structures a problem. The extraordinary visual and ergonomic advantages of the Da Vinci system were presumed to overcome the limitations of laparoscopy and improve the results of minimally invasive colon surgery [20]. Although the application of the Da Vinci system in the general field has increased year by year, due to the lack of effective tactile feedback assistance, intraoperative exploration is often difficult when the tumor is small or does not invade the serosa. It has been reported that the wrong colon segment is removed during laparoscopic surgery, which requires conversion to open surgery and resection of longer intestine segments [21, 22].

For colon tumors, colonoscopy is still the most sensitive diagnostic tool, but due to the lack of obvious anatomical landmarks in the colon, inaccurate tumor localization may lead to longer lengths of resection, and even the removal of normal intestinal segments leaving the tumor. 16.7% of cases will have different procedures from the original plan due to inaccurate preoperative colonoscopy, especially for transverse colon tumors [23]. So the 2013 Society of American Gastrointestinal and Endoscopic Surgeons (SAGES) and the 2004 European Association of Endoscopic Surgery (EAES) clinical practice guidelines for laparoscopic resection of colon cancer recommend colonoscopic tattooing of small lesions [24, 25]. But until now there were no evidence that suggest that colonoscopic tattooing should be used only for small lesions. Moreover, tumors localized in the transverse colon are commonly considered challenging, and routine tattooing of these tumors is recommended. Because the tumor is located close to the liver curvature, spleen curvature and the middle part of the transverse colon, the resection range is very different, so the surgical approach is divided into left hemicolectomy, right colon colectomy and transverse colon resection. In the Da Vinci surgical system, the three surgical methods, the placement of the robot and the port are different. If the surgical plan is changed during the operation, it will not only directly increase the cost of surgery, but also lead to prolonged operation time and increased risks of surgery. Therefore, how to achieve accurate preoperative and intraoperative positioning determines the premise of the success of robotic surgery.

There are a number of techniques currently used for the localization of colonic lesions, including double-contrast barium enema, computed tomography colonography, titanium clip positioning, intraoperative colonoscopy, and preoperative injection stain positioning. But each method has its shortcomings and limitations. Double-contrast barium enema and computed tomography colonography are easy to miss smaller lesions [12, 26]. Titanium clip positioning is a short-term solution, costly, often shifts or falls off after 2–3 weeks of implantation; and the titanium clip is small, the clip cannot be seen from the serosal side, and it cannot be touched during laparoscopic surgery [27]. Intraoperative colonoscopy can also be used for positioning, but it is a more complex method that requires experienced endoscopists and specific equipment in the operating room, which can increase the time of surgery and increase the risk of anesthesia and the probability of infection [7]. In addition, colonoscopy will inflate the intestines, increasing the difficulty of surgery [13]. In recent years, the most common method of positioning has been to inject a stain into the intestinal wall. Commonly used dyes such as methylene blue, indigo carmine, and phthalocyanine green have relatively short dyeing times, which tend to spread over time and contaminate the surgical field of view and cause inaccurate positioning [28, 29]. Although Indian inks have a long time at the marked parts, some studys reported Indian ink can cause peritonitis, cellulitis, gastritis, colonic abscesses and inflammatory pseudotumors [30–32].

The nano-carbon used in this study, Askin M P et al., evaluated the safety and efficacy of colon labeling using nano-carbon in a study of 113 patients [33]. In the study, no patients developed fever, abdominal pain or symptoms of inflammation, and nano-carbons existed for 1 year, confirming that nano-carbon is a long-term safe and effective marker. In this study, we also did not find any discomfort after the patient received nano-carbon injection. In addition, we use the "four quadrant" method [15] (ie, four points are selected centered on the tumor, each point is 90° apart) injection labeling is performed around the tumor to avoid staining of the intestinal wall when the tumor is on the mesenteric side. Moreover, the marker points are 1 cm away from the tumor and avoid direct injection into the tumor. Secondly, the needle is at an angle of 45° to the wall of the intestine when the needle is inserted, because the vertical needle easily penetrates the intestinal wall, causing the dye to enter the mesentery or the abdominal cavity to contaminate the surgical field of view. The diffusion of nano-carbon can also be reduced by the "three-step injection method" of J. W. Park et al. [34]. The final intraoperative findings showed that all 16 patients were able to find nano-carbon labeled sites, confirming that our approach worked. In this study, we placed a titanium clip on each of the anal side and the mouth side, and then immediately examined the radiation. The titanium clip showed a high signal in the X-ray, and the tumor was located between the two titanium clips. Studies have shown that two titanium clips are used to prevent displacement or shedding when using titanium clips for colon marking [27]. In general, the peak period of shedding is 2–3 weeks after placement, the longer the time, the greater the probability of shedding. Radiation inspection immediately after the titanium clip is placed can reduce errors caused by displacement or shedding of the titanium clip. Moreover, all patients underwent surgery within 1 week after receiving the marker, thereby avoiding the peak of titanium clip detachment.

Nano-carbon labeling helps us to quickly find tumors during surgery, avoid excision of the wrong bowel segment, and ensure a sufficient safety margin. Titanium clip marking allows us to obtain a more accurate positioning before surgery, which helps to develop a surgical plan and avoid the cost associated with robotic surgery and additional operative time due to changes to the surgical plan. Accurate preoperative positioning can provide a reliable basis for the selection of Trocar position and surgical incision for laparoscopic surgery, to avoid surgical errors due to poor exposure of the surgical field caused by incorrect selection of Trocar position. In general, robotic surgery has a longer operation time than laparoscopic surgery. Although long operation time may be related to high postoperative morbidity, operative time is not the only parameter showing the quality of surgery and it is obvious that the operation time may decrease as the experience of robotic surgery increases. And our method can significantly reduce the exploration time.

## Conclusion

In conclusion, the findings of this study have shown that the preoperative colonoscopy nano-carbon and titanium clip combined labeling method is safe and effective in robot-assisted transverse colon cancer surgery. At the same time, the labeling method shows potential in shortening the operation time, ensuring sufficient safety margin and reducing complications.

## Data Availability

The datasets used and analysed during the current study are available from the corresponding author on reasonable request.

## References

[1] Ferlay J, Soerjomataram I, Dikshit R, Eser S, Mathers C, Rebelo M, Parkin DM, Forman D, Bray F (2015). Cancer incidence and mortality worldwide: sources, methods and major patterns in globocan 2012. Int J Cancer.

[2] Chen W, Zheng R, Zeng H, Zhang S (2014). The incidences and mortalities of major cancers in China 2010. Chin J Cancer.

[3] Antoniou SA, Antoniou GA, Koch OO, Pointner R, Granderath FA (2012). Robot-assisted laparoscopic surgery of the colon and rectum. Surg Endosc.

[4] Bartels SA, Vlug MS, Hollmann MW, Dijkgraaf MG, Ubbink DT, Cense HA, van Wagensveld BA, Engel AF, Gerhards MF, Bemelman WA (2014). Small bowel obstruction, incisional hernia and survival after laparoscopic and open colonic resection (LAFA study). Br J Surg.

[5] Bernasconi M, Metzger J (2010). Randomized clinical trial comparing laparoscopic and open surgery in patients with rectal cancer. Br J Surg.

[6] Kouhia ST, Heiskanen JT, Huttunen R, Ahtola HI, Kiviniemi VV, Hakala T (2010). Long-term follow-up of a randomized clinical trial of open versus laparoscopic appendicectomy. Br J Surg.

[7] Zmora O, Dinnewitzer AJ, Pikarsky AJ, Efron JE, Weiss EG, Nogueras JJ (2002). Intraoperative endoscopy in laparoscopic colectomy. Surg Endosc.

[8] Halligan S, Wooldrage K, Dadswell E, Kralj-Hans I, von Wagner C, Edwards R, Yao G, Kay C, Burling D, Faiz O, Teare J, Lilford RJ, Morton D, Wardle J, Atkin W (2013). Computed tomographic colonography versus barium enema for diagnosis of colorectal cancer or large polyps in symptomatic patients (SIGGAR): a multicentre randomised trial. The Lancet.

[9] Gorgun IE, Aytac E, Manilich E, Church JM, Remzi FH (2013). Intraoperative colonoscopy does not worsen the outcomes of laparoscopic colorectal surgery: a case-matched study. Surg Endosc.

[10] Yeung JMC, Maxwell-Armstrong C, Acheson AG (2010). Colonic tattooing in laparoscopic surgery—making the mark?. Colorectal Dis.

[11] Yan J, Xue F, Chen H, Wu X, Zhang H, Chen G, Lu J, Cai L, Xiang G, Deng Z, Zheng Y, Zheng X, Li G (2014). A multi-center study of using carbon nanoparticles to track lymph node metastasis in t1–2 colorectal cancer. Surg Endosc.

[12] Rockey DC, Paulson E, Niedzwiecki D, Davis W, Bosworth HB, Sanders L, Yee J, Henderson J, Hatten P, Burdick S, Sanyal A, Rubin DT, Sterling M, Akerkar G, Bhutani MS, Binmoeller K, Garvie J, Bini EJ, McQuaid K, Foster WL, Thompson WM, Dachman A, Halvorsen R (2005). Analysis of air contrast barium enema, computed tomographic colonography, and colonoscopy: prospective comparison. Lancet.

[13] Wang W, Wang R, Wang Y, Yu L, Li D, Huang S, Ma J, Lin N, Yang W, Chen X, Liu B, Lv R, Liao L (2013). Preoperative colonic lesion localization with charcoal nanoparticle tattooing for laparoscopic colorectal surgery. J Biomed Nanotechnol.

[14] Jiang Y, Lin N, Huang S, Lin C, Jin N, Zhang Z, Ke J, Yu Y, Zhu J, Wang Y (2015). Tracking nonpalpable breast cancer for breast-conserving surgery with carbon nanoparticles: implication in tumor location and lymph node dissection. Medicine.

[15] Hyman N, Waye JD (1991). Endoscopic four quadrant tattoo for the identification of colonic lesions at surgery. Gastrointest Endosc.

[16] Hashizume M, Shimada M, Tomikawa M, Ikeda Y, Takahashi I, Abe R, Koga F, Gotoh N, Konishi K, Maehara S, Sugimachi K (2002). Early experiences of endoscopic procedures in general surgery assisted by a computer-enhanced surgical system. Surv Methodol.

[17] Weber PA, Merola S, Wasielewski A, Ballantyne GH (2002). Telerobotic-assisted laparoscopic right and sigmoid colectomies for benign disease. Dis Colon Rectum.

[18] Kim CW, Kim CH, Baik SH (2014). Outcomes of robotic-assisted colorectal surgery compared with laparoscopic and open surgery: a systematic review. J Gastrointest Surg.

[19] Lin S, Jiang HG, Chen ZH, Zhou SY, Liu XS, Yu JR (2011). Meta-analysis of robotic and laparoscopic surgery for treatment of rectal cancer. World J Gastroenterol.

[20] Gorgun E, Aytac E, Gurland B, Costedio MM (2015). Case-matched comparison of robotic versus laparoscopic colorectal surgery: initial institutional experience. Surg Laparosc Endosc Percutan Tech.

[21] Wishner JD, Baker JW, Hoffman GC, Hubbard GW, Gould RJ, Wohlgemuth SD, Ruffin WK, Melick CF (1995). Laparoscopic-assisted colectomy—the learning curve. Surg Endosc.

[22] Wexner SD, Cohen SM, Ulrich A, Reissman P (1995). Laparoscopic colorectal surgery - are we being honest with our patients?. Dis Colon Rectum.

[23] Fernandez LM, Ibrahim RNM, Mizrahi I, DaSilva G, Wexner SD (2019). How accurate is preoperative colonoscopic localization of colonic neoplasia?. Surg Endosc.

[24] Veldkamp R, Gholghesaei M, Bonjer HJ, Meijer DW, Buunen M, Jeekel J, Anderberg B, Cuesta MA, Cuschierl A, Fingerhut A, Fleshman JW, Guillou PJ, Haglind E, Himpens J, Jacobi CA, Jakimowicz JJ, Koeckerling F, Lacy AM, Lezoche E, Monson JR, Morino M, Neugebauer E, Wexner SD, Whelan RL (2004). Laparoscopic resection of colon cancer: consensus of the european association of endoscopic surgery (EAES). Surg Endosc.

[25] Zerey M, Hawver LM, Awad Z, Stefanidis D, Richardson W, Fanelli RD (2013). Sages evidence-based guidelines for the laparoscopic resection of curable colon and rectal cancer. Surg Endosc.

[26] Atkin W, Dadswell E, Wooldrage K, Kralj-Hans I, von Wagner C, Edwards R, Yao G, Kay C, Burling D, Faiz O, Teare J, Lilford RJ, Morton D, Wardle J, Halligan S (2013). Computed tomographic colonography versus colonoscopy for investigation of patients with symptoms suggestive of colorectal cancer (SIGGAR): a multicentre randomised trial. The Lancet.

[27] Ohdaira T, Konishi F, Nagai H, Kashiwagi H, Shito K, Togashi K, Kanazawa K (1999). Intraoperative localization of colorectal tumors in the early stages using a marking clip detector system. Dis Colon Rectum.

[28] Price N, Gottfried MR, Clary E, Lawson DC, Baillie J, Mergener K, Westcott C, Eubanks S, Pappas TN (2000). Safety and efficacy of india ink and indocyanine green as colonic tattooing agents. Gastrointest Endosc.

[29] Committee AT, Kethu SR, Banerjee S (2010). Endoscopic tattooing. Gastrointest Endosc.

[30] Hornig D, Kühn H, Stadelmann O, Bötticher R (1983). Phlegmonous gastritis after India Ink marking. Endoscopy.

[31] Park SI, Genta RS, Romeo DP, Weesner RE (1991). Colonic abscess and focal peritonitis secondary to india ink tattooing of the colon. Gastrointest Endosc.

[32] Coman E, Brandt LJ, Brenner S, Frank M, Sablay B, Bennett B (1991). Fat necrosis and inflammatory pseudotumor due to endoscopic tattooing of the colon with india ink. Gastrointest Endosc.

[33] Askin MP, Waye JD, Fiedler L, Harpaz N (2002). Tattoo of colonic neoplasms in 113 patients with a new sterile carbon compound. Gastrointest Endosc.

[34] Park JW, Sohn DK, Hong CW, Han KS, Choi DH, Chang HJ, Lim SB, Choi HS, Jeong SY (2008). The usefulness of preoperative colonoscopic tattooing using a saline test injection method with prepackaged sterile india ink for localization in laparoscopic colorectal surgery. Surg Endosc.

